# Improving Perioperative Tranexamic Acid Administration in Hip Fracture Surgery: A Two-Cycle Quality Improvement Project at Dorset County Hospital

**DOI:** 10.7759/cureus.98220

**Published:** 2025-12-01

**Authors:** Mohamed E Mahmoud, Qamar Mustafa, Owen Mitchell

**Affiliations:** 1 Trauma and Orthopaedics, Dorset County Hospital NHS Foundation Trust, Dorchester, GBR

**Keywords:** fragility hip fracture, perioperative blood loss, perioperative medicine, quality improvement research, tranexamic acid (txa)

## Abstract

Background

Tranexamic acid (TXA) is widely used in surgical practice to reduce bleeding by inhibiting fibrinolysis. Despite its established role, utilisation in hip fracture surgery varies across institutions. This quality improvement project evaluated perioperative TXA administration within a district general hospital and assessed the impact of targeted interventions on prescribing practices.

Methods

A quality improvement project was initiated at Dorset County Hospital (DCH), Dorchester, England, to evaluate the utilisation of TXA in patients admitted with hip fractures that were surgically treated. All patients aged 65 and over undergoing surgical management of hip fractures were included, excluding those with contraindications (recent thromboembolic events, active clotting disorders, hypersensitivity, or recent stenting). Two audit cycles were conducted, i.e., September-November 2021 and September-October 2023, assessing perioperative TXA administration rates.

Results

TXA use increased from 70% (n = 37) in the first cycle to 85.2% (n = 27) in the second cycle. Improved uptake correlated with enhanced staff awareness and adherence to updated local protocols.

Conclusion

Implementing clear local guidelines and ensuring documentation of contraindications significantly improved TXA administration in hip fracture surgery. These measures supported more consistent practice and contributed to an overall improvement in perioperative care within the department.

## Introduction

Hip fractures are one of the most common orthopaedic presentations in the United Kingdom, with over 70,000 cases annually [1,2]. They carry a one-year mortality of around 30% and a substantial burden on patients, families, and health systems [3]. This high incidence highlights the critical need for effective interventions to optimize perioperative care and minimize complications such as perioperative anaemia and prolonged admissions that are associated with these procedures.

While surgical intervention is the mainstay of treatment, perioperative bleeding remains a major challenge. Both visible and hidden blood loss can exceed one litre, often leading to perioperative anaemia and high rates of allogeneic red blood cell transfusion [4]. Transfusion, while sometimes lifesaving, is associated with longer hospital stay, infection risk, immunological complications, and increased costs, particularly in frail older adults [5]. 

Tranexamic acid (TXA) is a low-cost antifibrinolytic agent with a proven ability to reduce surgical bleeding by stabilising fibrin clots. It works by inhibiting the activation of plasminogen to plasmin, thus preventing the breakdown of fibrin [6]. This mechanism is particularly beneficial in hip fracture surgeries, where maintaining hemostasis is crucial for optimal recovery and minimizing complications [2]. Its efficacy and safety profile are well documented across trauma, obstetrics, and elective orthopaedic surgery [7]. Importantly, the Clinical Randomisation of an Antifibrinolytic in Significant Head injury (CRASH)-2 and World Maternal Antifibrinolytic (WOMAN) trials showed significant survival benefits in major haemorrhage, without excess thromboembolic events [8,9]. In the context of hip fracture surgery, systematic reviews have consistently shown TXA to reduce transfusion requirements without increasing venous thromboembolism [10].

Despite this strong evidence, real-world uptake remains inconsistent. The Perioperative Administration of Tranexamic acid in Hip Fracture Surgery (PATHS) study highlighted that only 48% (428 out of 891) of eligible hip fracture patients received TXA [2]. It has also shown that the administration rates varied significantly across institutions, emphasizing the need for improved adherence to established protocols and guidelines for TXA use in hip fracture surgeries. The National Institute for Health and Care Excellence (NICE) guideline explicitly recommends offering TXA to adults undergoing surgery expected to result in moderate blood loss, defined as greater than 500 millilitres [1]. This threshold is clearly exceeded in most hip fracture surgeries, yet barriers such as lack of awareness, inconsistent protocols, and concerns about thrombotic risk continue to limit implementation.

This quality improvement project aimed to address these gaps at Dorset County Hospital (DCH) in Dorchester, England, by assessing baseline practice, implementing targeted interventions, and re-auditing outcomes. The goal was to ensure consistent perioperative TXA administration in line with national guidance, while safeguarding patients through rigorous documentation of contraindications.

## Materials and methods

All patients aged 65 or older undergoing surgical management of hip fracture at DCH were included, unless contraindications to TXA were present. Contraindications included recent thromboembolic events, active clotting disorders, hypersensitivity, or recent coronary stenting in the last six months.

Two audit cycles were performed. The first, between September and November 2021, measured baseline practice. Following this, interventions included updating local hip fracture protocols to include TXA administration, targeted teaching sessions for anaesthetic and theatre staff, and encouraging the recording of contraindications. The second cycle, conducted in September and October 2023, assessed the impact of these changes.

The primary outcome was the proportion of eligible patients receiving TXA perioperatively. Secondary outcomes included documentation of contraindications and any observed increase in thromboembolic complications.

## Results

**Figure 1:**
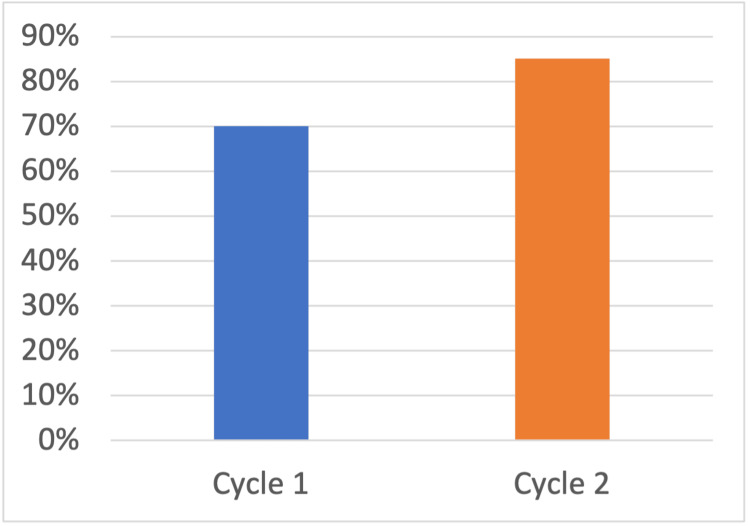
Percentage of eligible patients who received TXA in both cycles

In the first audit cycle, 37 eligible patients were identified. Of this cohort, 26 (70%) received TXA intraoperatively. There were no documented contraindications of thromboembolic complications. The education programme was then delivered, and the audit was repeated. The second cycle included 27 eligible patients, of whom 23 received intraoperative TXA. This shows an increase of 15.2% (see Figure 1). Documentation of contraindications also improved, providing greater clarity around reasons for omission (Table 1). Importantly, there was no increase in documented thromboembolic events between cycles.

**Table 1:** Outcomes of tranexamic acid (TXA) administration between audit cycles

Outcome	Cycle 1 (Sep–Nov 2021)	Cycle 2 (Sep–Oct 2023)
Eligible patients (n)	37	27
TXA administered (n)	26	23
TXA administered (%)	70.0%	85.2%
Contraindication documented (%)	0%	7.4%
Thromboembolic complications (n)	0	0

Using Fisher’s exact test, this difference was not statistically significant (p = 0.23, odds ratio = 0.41) (see Table 2). Although not statistically significant at the 5% level, the observed increase indicates a clinically relevant improvement in adherence to TXA administration protocols following the implemented interventions.

**Table 2:** Fisher's exact test analytic results n= number of patients eligible for TXA administration, P value (two-tailed) = 0.2347, odds ratio = 0.41

	TXA administered (n)	TXA not administered (n)	Total (n)
First cycle (n)	26	11	37
Second cycle (n)	23	4	27
Total (n)	49	15	64

## Discussion

This quality improvement project demonstrates that simple, targeted interventions can meaningfully change clinical practice in hip fracture surgery. The increase from 70.0% (n = 37) to 85.2% (n = 27) TXA administration represents not only a local improvement but also a performance level substantially higher than the national average. The final figures compared favorably with the national PATHS study, which reported TXA use in only 48% (428 out of 891) of eligible cases [2]. Although the increase in TXA administration between audit cycles was not statistically significant (p = 0.23, Fisher’s exact test), the trend represents a clinically meaningful improvement in compliance with national guidance and PATHS study recommendations.

The fact that this improvement was achieved without any increase in thromboembolic complications should reassure clinicians that TXA is safe when used according to evidence-based protocols. The findings also highlight the gap between robust evidence and clinical implementation. To bridge this gap, ongoing education and adherence to established guidelines are essential for promoting the safe use of TXA in hip fracture surgeries.

TXA has been available for decades, is inexpensive, and has consistently shown benefit in reducing bleeding across multiple surgical and trauma contexts [7-9]. However, barriers such as variable clinician awareness, inertia in local protocols, and fears about thrombotic complications persist [11]. The interventions at DCH, particularly education and clear documentation prompts, addressed these barriers directly. Importantly, improved documentation of contraindications not only increased confidence in prescribing TXA but also ensured patient safety, providing transparency when the drug was appropriately withheld.

From a health system perspective, optimising TXA use has wider implications. Transfusion avoidance reduces costs, conserves limited blood supplies, and reduces transfusion-related morbidity [4]. Current meta-analysis supports the use of TXA in hip fracture surgery, demonstrating significant reductions in total blood loss and transfusion rates. For example, a recent meta-analysis of 18 studies (14 RCTs) found that TXA reduced total blood loss and blood transfusion odds without increasing thromboembolic events [12]. Another meta-analysis from 2023 similarly concluded that TXA significantly lowered intraoperative and overall blood loss, transfusion rate, and maintained higher haemoglobin levels post-op, with no significant increase in DVT/PE or mortality [13]. In frail hip fracture patients, avoiding anaemia and transfusion may shorten recovery times, facilitate earlier mobilisation, and reduce length of stay. While these outcomes were not directly measured in this project, existing literature supports such benefits [10,11]. Future audit cycles at DCH should extend beyond process measures to evaluate transfusion rates, length of stay, functional outcomes, and 30-day morbidity.

This project does have limitations. It was observational in design, dependent on accurate documentation, and not powered to detect rare adverse events. The absence of direct transfusion data restricts conclusions about downstream patient outcomes. Also, given the relatively small sample size, the analysis was likely underpowered to detect statistical significance. Nevertheless, the relative improvement in TXA administration demonstrates that quality improvement methods can rapidly align practice with guidelines and national standards. Furthermore, it does not encompass the functional outcomes for patients who received TXA in comparison to those who did not. Future cycles should focus on the long-term effects of TXA administration on patient outcomes, including functional recovery and overall quality of life post-surgery.

Overall, this work reinforces the importance of embedding evidence-based interventions within local practice through education, audit, and feedback. It also demonstrates how quality improvement can be a pragmatic bridge between national policy and day-to-day patient care. Further efforts should focus on sustaining these improvements through ongoing education and regular audits to ensure continued adherence to TXA guidelines in hip fracture surgeries for improved recovery and reducing complications.

## Conclusions

This audit has shown that the implementation of more specific local protocols, coupled with heightened staff awareness and enhanced documentation practices, has improved the utilisation of TXA in hip fracture surgeries at Dorset County Hospital. This focused initiative has led to an increase in the rate of TXA administration, which surpasses national averages and is in line with the NICE guidelines.

These enhancements are expected to play a crucial role in reducing the necessity for blood transfusions, thereby promoting greater patient safety and optimising recovery outcomes. The proactive measures taken reflect a commitment to evidence-based medicine, ensuring that patients receive the best possible care during their surgical interventions.

To further solidify these advancements, Further audits should encompass both transfusion metrics and various clinical endpoints, ultimately leading to improved patient outcomes and a more efficient surgical process.
